# Genetic and causal relationship between chronic gastrointestinal diseases and erectile dysfunction: a Mendelian randomization study

**DOI:** 10.3389/fmed.2024.1422267

**Published:** 2024-07-31

**Authors:** Xiaoyan Zeng, Li Tong

**Affiliations:** ^1^Qinghai University, Xining, China; ^2^Qinghai Provincial Key Laboratory of Traditional Chinese Medicine Research for Glucolipid Metabolic Diseases, Xining, China; ^3^The Central Hospital of Enshi Tujia and Miao Autonomous Prefecture, Enshi City, China

**Keywords:** Mendelian randomization, chronic gastrointestinal diseases, erectile dysfunction, inflammatory bowel disease, colorectal cancer

## Abstract

**Background:**

Studies based on observations have indicated potential associations between chronic gastrointestinal diseases and an increased risk of erectile dysfunction (ED). However, the causality of these connections remains ambiguous.

**Methods:**

Summary data for chronic gastrointestinal diseases were extracted from public data. Summary data on ED were extracted from three distinct sources. The genetic correlations between chronic gastrointestinal diseases and ED were explored using linkage disequilibrium score regression (LDSC). The causal associations between chronic gastrointestinal diseases and ED were evaluated using Mendelian randomization (MR) analysis, followed by a meta-analysis to determine the ultimate causal effect.

**Results:**

The LDSC results suggested significant genetic correlations between Crohn's disease (CD) and ED. Inflammatory bowel disease (IBD), ulcerative colitis (UC), and liver cirrhosis (LC) were found to have potential genetic correlations with ED. The combined multiple MR results indicate that IBD and CD have significant causal relationships with ED, while colorectal cancer (CRC) may have a potential causal effect on ED.

**Conclusion:**

This research provided evidence supporting a causal association between IBD, CD, CRC, and ED. The impact of chronic gastrointestinal diseases on ED warrants greater attention in clinical practice.

## 1 Introduction

Erectile dysfunction (ED) is characterized by the inability to attain and sustain a satisfactory penile erection (1, 2). According to Goldstein et al.'s research, the prevalence of ED varied among nations, with rates ranging from 37.2% to 48.6%, while a recent study indicated that the prevalence of ED in younger males was approximately 30% (3, 4). ED shows a robust correlation with several chronic conditions, including chronic gastrointestinal diseases. An observation-based investigation emphasized a significant connection between chronic gastrointestinal diseases and ED, although the exact cause-and-effect relationship remains uncertain (5).

According to reports, chronic gastrointestinal diseases hurt male sexual function; however, the exact mechanism remains ambiguous (6). Chronic gastrointestinal diseases may trigger inflammatory responses in the body, which can disrupt normal physiological processes and potentially lead to ED. Psychological stress arising from chronic gastrointestinal diseases might impact sexual function. Additionally, changes in endocrine levels, such as sex hormones, could result in genital dysfunction (5). Epidemiological research has found that chronic gastrointestinal diseases may increase the incidence of ED. Nevertheless, the current research displays inadequacies that hinder the formation of convincing evidence for these associations. Clarifying the causal links between chronic gastrointestinal diseases and ED is vital for developing targeted prevention strategies and improving patient outcomes. Unmeasurable confounders, like diabetes, and reverse causation make these causal associations difficult to clarify (7). Mendelian randomization (MR) has emerged as a dependable tool in epidemiological research for determining causality (8).

The use of MR in epidemiological research is an emerging methodology, using genetic variants as instrumental variables (IVs) to establish causal associations between exposures and outcomes (9, 10). Compared with observational studies, MR is adept at alleviating the impact of confounders and reverse causation (11). In addition, MR can effectively save financial and human resource costs (12). Several genome-wide association studies (GWAS) provide dependable IVs in MR studies by using genetic variations. To mitigate the influence of environmental factors, alleles adhere to the principle of independent assortment, resembling the structure of randomized controlled trials (RCTs) (13, 14).

Thus, a two-sample MR analysis was conducted to comprehensively investigate the causality of chronic gastrointestinal diseases [chronic gastritis (CG), peptic ulcer disease (PUD), gastric cancer (GC), inflammatory bowel disease (IBD) including Crohn's disease (CD) and ulcerative colitis (UC), celiac disease (CeD), irritable bowel syndrome (IBS), nonalcoholic fatty liver disease (NAFLD), colorectal cancer (CRC), alcoholic liver disease (ALD), chronic pancreatitis (CP), liver cirrhosis (LC)] on ED.

## 2 Materials and methods

### 2.1 Research design

**Figure 1:**
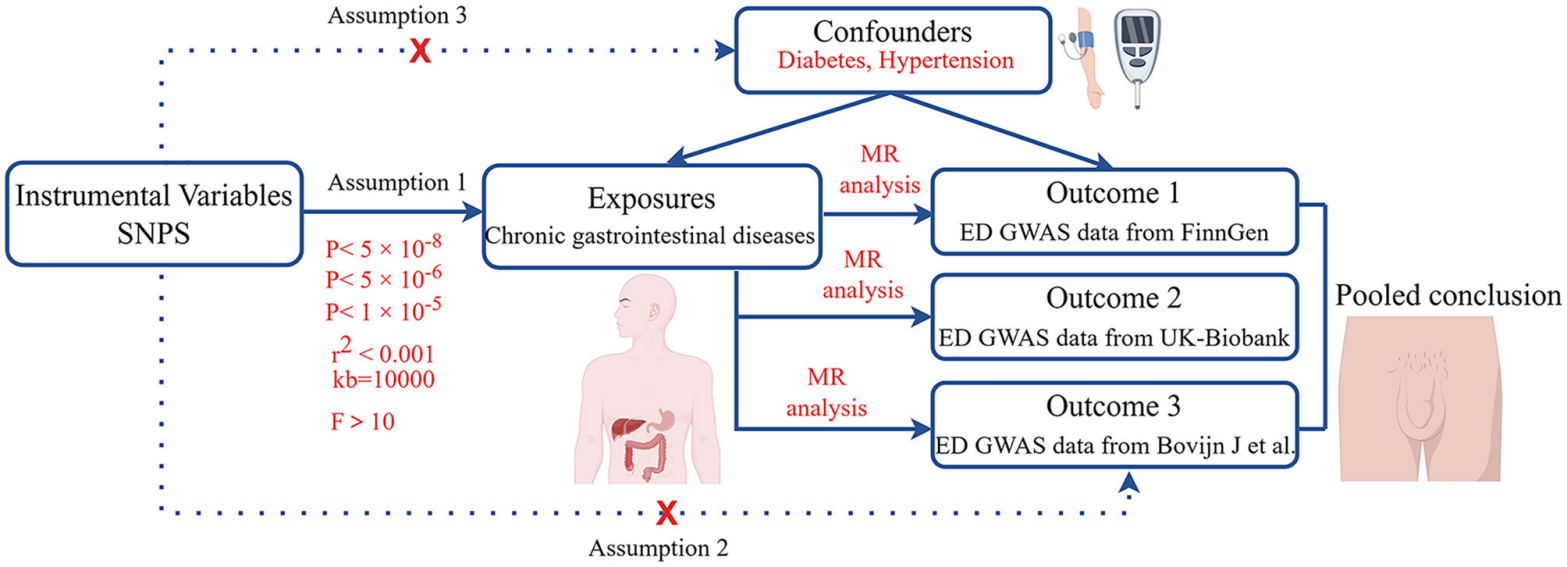
Workflow of this MR study. ED, erectile dysfunction; MR, Mendelian randomization.

Initially, we calculated the genetic correlations between chronic gastrointestinal diseases and ED. Subsequently, the causal relationships between chronic gastrointestinal diseases and ED were explored using MR analysis, by analyzing data from the FinnGen study (Figure 1). Then, we performed replication studies using the UK Biobank data on ED and additional GWAS summary data (Figure 1). Ultimately, we integrated the results from these MR studies (Figure 1). The single nucleotide polymorphism (SNP) used in our research must adhere to three key assumptions: 1. Close connection of the exposure to the IV is essential. 2. The outcome is directly influenced by the IV primarily through exposure. 3. The IV should demonstrate no correlation with potential confounding factors (15, 16). Our GWAS data summaries sourced from published GWAS research, did not require ethics committee approval.

### 2.2 GWAS summary data sources

The IEU OpenGWAS project has provided summary data for CG, GC, LC, and CP (17), including 3,645 CG patients (441,451 controls), 1029 GC patients (475,087 controls), 122 LC patients (347,284 controls), and 1,424 CP patients (476,104 controls). The summary statistical data of PUD was sourced from a genome-wide association study (920 cases, 55,717 controls) (17, 18). The International Inflammatory Bowel Disease Genetics Consortium (IIBDGC) provided IBD, UC, and CD GWAS data. The data included 21,770 controls and 12,882 IBD cases, 14,927 controls, and 5,956 CD cases, as well as 33,977 controls and 13,768 UC cases (19). Summary data from a published GWAS study yielded 12,041 cases and 12,228 controls for Ced (20). Another GWAS study by Eijsbouts C et al. contributed summary data for IBS, comprising 53,400 cases and 433,201 controls (21). Additionally, a GWAS analysis on CRC (11,835 patients, 11,856 controls) offered data for CRC (22). The NAFLD summary data was from a GWAS analysis (8,434 cases, 770,180 controls) (23). The summary data for ALD (451 cases and 455,897 controls) was derived from analyses of UK Biobank data by Jiang et al. (24).

In the discovery stage, we chose the FinnGen consortium's data on ED, comprising 1,154 cases and 94,024 controls. For replication stage 1, the study used summary data for ED in the UK Biobank study (357 patients and 208,451 controls) (24). Additionally, data from a GWAS study on ED, with 6,175 cases and 217,360 controls, was selected for replication stage 2 (25). ED was defined as self-reported or physician-reported ED using ICD10 codes N48.4 and F52.2, or use of oral ED medication, or a history of surgical intervention for ED (using OPCS-4 codes L97.1 and N32.6). All participants were reported to have European ancestry. Supplementary Table 1 contains details of the data sources.

### 2.3 Genetic instruments selection

To ensure high-quality SNPs, we followed rigorous procedures. SNPs associated with IBD, CD, CeD, and UC were identified at *P* < 5 × 10^−8^. SNPs demonstrating a strong association with CP were selected at *P* < 5 × 10^−6^ (26). *P* < 1 × 10^−5^ was employed for CG, PUD, GC, IBS, CRC, NAFLD, LC, and ALD (27). To prevent linkage disequilibrium, a stringent criterion was implemented, setting kb at 10,000 and requiring r^2^ to be < 0.001 (28). During the harmonization process, palindromic and incompatible alleles were systematically excluded. To prevent potential pleiotropic effects, SNPs associated with confounding factors like diabetes and hypertension were removed using PhenoScanner V2 (29). Moreover, to minimize weak instrumental variable bias, we specifically chose SNPs with *F* statistic >10 (*F* = *Beta*^2^/*SE*^2^) for MR study (30). Supplementary Tables 2–4 contain details of the SNPs.

### 2.4 Statistical analysis

The genetic correlations between chronic gastrointestinal diseases and ED were assessed using linkage disequilibrium score regression (LDSC). LDSC calculated genetic correlation using GWAS data, unaffected by sample overlap (31, 32). The correlation is represented as genetic covariance normalized by SNP heritability (33). Causal associations between chronic gastrointestinal diseases and ED were primarily evaluated by the inverse variance weighted (IVW) method (9, 34). The sensitivity analyses included weighted median (WM), MR-Egger, weighted mode, and simple mode. Reliable causal estimates can be provided by the WM method when over half of the weight stems from valid IVs (35). Despite the presence of pleiotropy in the IVs, the MR-Egger approach can still produce reliable estimates (36). The weighted model is considered valid for conducting causal inference in the MR study (37). A less powerful alternative, the simple mode method, was also employed in our study (38). Heterogeneity among SNPs was assessed through Cochrane's Q test, whereas horizontal pleiotropy was examined by analyzing the intercept in MR-Egger analysis (39). We used MR-PRESSO analysis to help identify potential outliers that could impact the results and then refine the causal estimates by excluding these outlying instrumental variables (36). The SNPs showed no significant heterogeneity or pleiotropy if the *P* values were above 0.05. A leave-one-out analysis was conducted to enhance the accuracy of causal estimates (40). Finally, we used the meta-analysis to explore the final causality, by integrating the initial discovery phase and the subsequent replication stage results (41). A fixed-effects model was chosen for I^2^ values ≤ 50%, while a random-effects model was used for I^2^ values >50%.

In this study, a rigorous Bonferroni correction was implemented. *P* < 0.0038 (0.05/13) indicated significant causal relationships between chronic gastrointestinal diseases and ED. *P* values ranging from 0.0038 to 0.05 suggested possible causation. The statistical analysis was conducted with the TwoSampleMR and MR-PRESSO (36, 40) packages in R version 4.3.2.

## 3 Results

### 3.1 Genetic correlation

**Figure 2:**
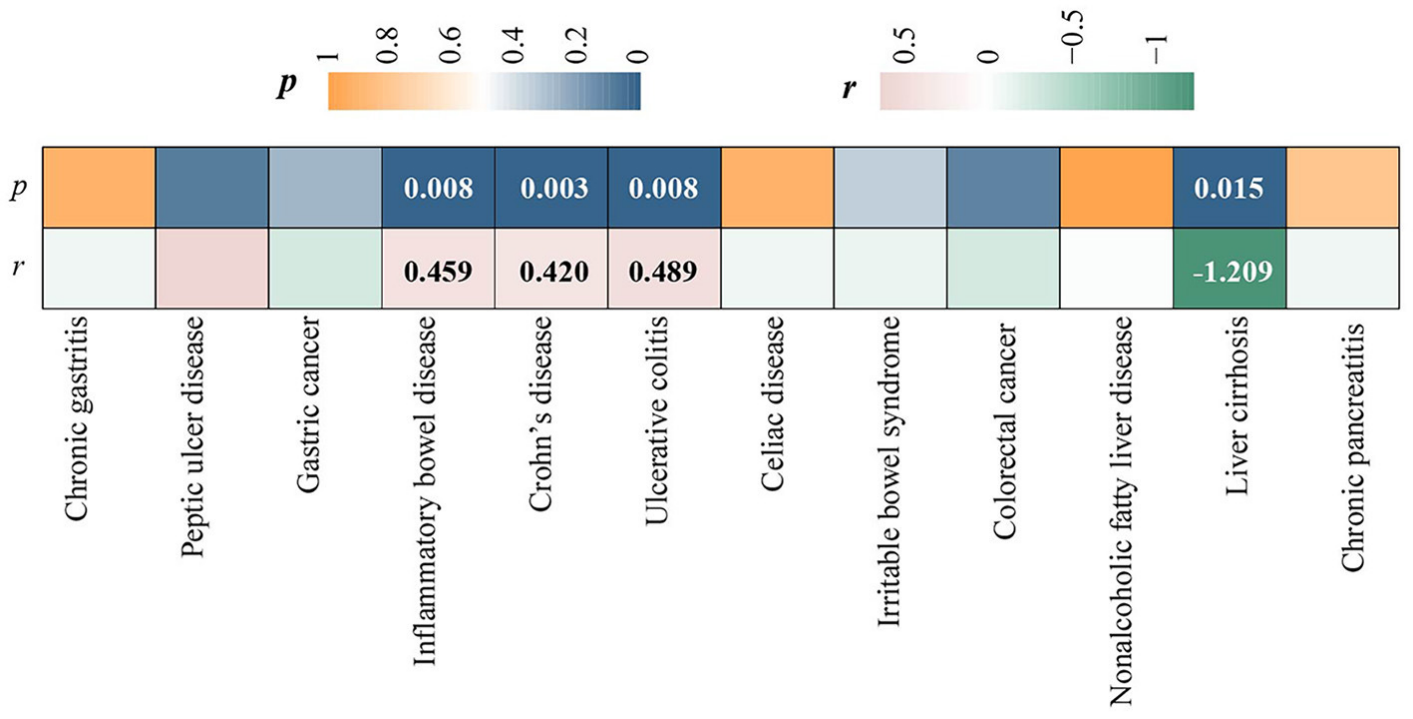
Heatmap of genetic correlations between chronic gastrointestinal diseases and ED.

LDSC regression analysis was conducted using FinnGen data. Due to the limitation of low heritability, ALD was not included in the above analysis. We observed a significant forward genetic correlation between CD and ED (*r*_*g*_= 0.420, *p* = 0.003) (Figure 2). IBD (*r*_*g*_= 0.420, *p*=0.008) and UC (*r*_*g*_= 0.489, *p* = 0.008) exhibited potential forward genetic correlations with ED (Figure 2). Furthermore, a negative suggestive genetic correlation was identified between LC and ED (*r*_*g*_= −1.209, *p* = 0.015) (Figure 2).

### 3.2 Discovery results

**Figure 3:**
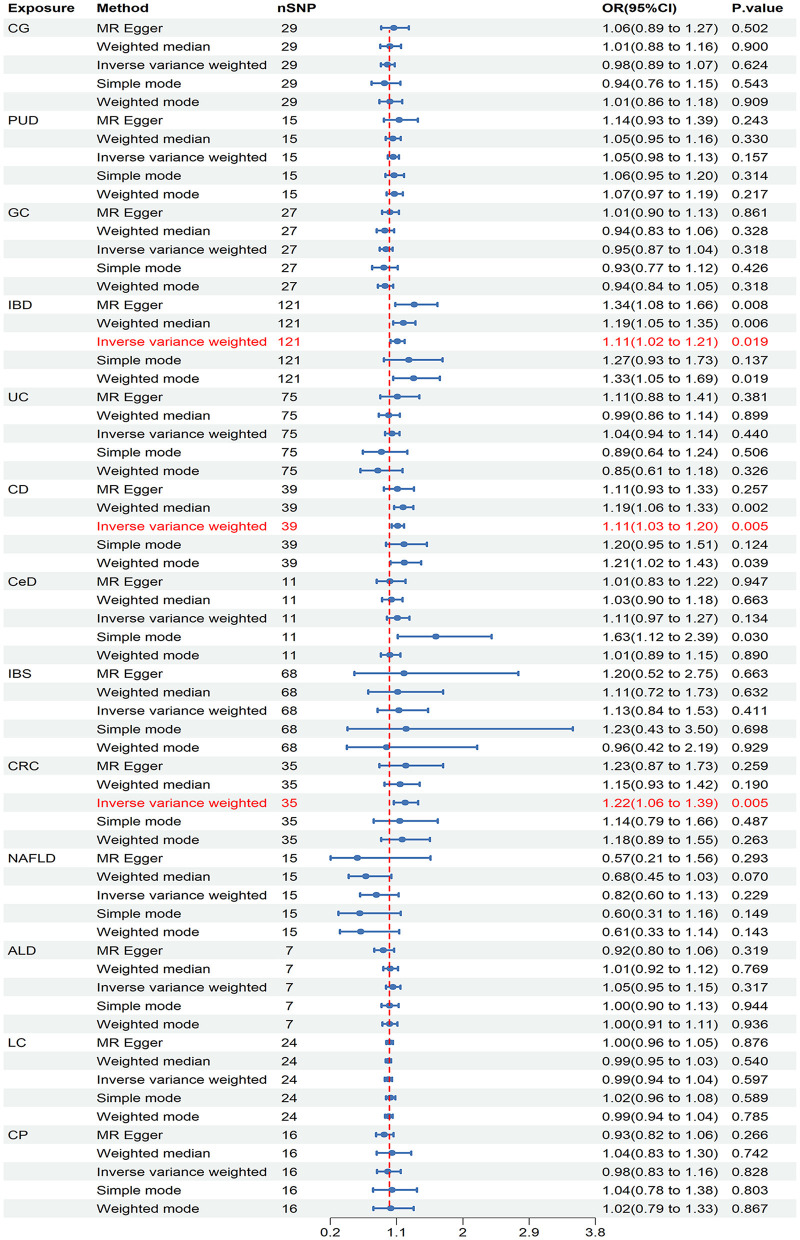
Causal estimates of chronic gastrointestinal diseases on ED in FinnGen. CI, confidence interval; CG, chronic gastritis; PUD, peptic ulcer disease; GC, gastric cancer; IBD, inflammatory bowel disease; UC, ulcerative colitis; CD, Crohn's disease; CeD, celiac disease; IBS, irritable bowel syndrome; CRC, colorectal cancer; NAFLD, nonalcoholic fatty liver disease; ALD, alcoholic liver disease; LC, liver cirrhosis; CP, chronic pancreatitis.

In the discovery stage, it was observed that IBD (OR = 1.110, 95% CI = 1.017–1.211, *P* = 0.019), CD (OR = 1.114, 95% CI = 1.034–1.201, *P* = 0.005), and CRC (OR = 1.216, 95% CI = 1.062–1.393, *P* = 0.005) may have potential associations with ED (Figure 3). Significant heterogeneity was not observed in the Cochran's Q test in this study (Table 1). The MR-Egger intercept test and MR-PRESSO global tests revealed no evidence of pleiotropy (Table 1). Furthermore, there were no outliers found, and the robustness of the findings was additionally confirmed through the leave-one-out examination.

**Table 1:** Heterogeneity and pleiotropy test.

**Exposure**	**Data sources**	**Cochran's Q**		**MR Egger**		**MR PRESSO**	
		**Q**	***P***	**Intercept**	***P***	**Global test RSSobs**	***P***
CG	FinnGen	22.590	0.753	−0.023	0.267	24.413	0.760
	UK Biobank	4.166	0.842	−0.002	0.972	5.402	0.854
	Bovijn J et al.	37.292	0.113	−0.010	0.376	40.141	0.105
PUD	FinnGen	10.034	0.760	−0.039	0.453	11.290	0.792
	UK Biobank	5.802	0.326	0.194	0.258	7.332	0.430
	Bovijn J et al.	11.240	0.591	0.006	0.802	12.827	0.647
GC	FinnGen	21.319	0.725	−0.027	0.125	22.349	0.759
	UK Biobank	12.046	0.524	0.038	0.443	13.455	0.567
	Bovijn J et al.	16.359	0.979	0.003	0.757	17.369	0.978
IBD	FinnGen	138.330	0.121	−0.022	0.058	140.883	0.118
	UK Biobank	35.717	0.777	0.008	0.751	37.038	0.789
UC	FinnGen	80.056	0.295	−0.009	0.533	82.836	0.278
	UK Biobank	13.437	0.991	0.013	0.715	15.002	0.985
CD	FinnGen	32.804	0.708	0.000	0.984	34.844	0.694
	UK Biobank	8.998	0.773	−0.002	0.965	9.900	0.807
CeD	FinnGen	17.241	0.069	0.041	0.202	21.513	0.101
	UK Biobank	3.234	0.779	−0.088	0.145	3.716	0.848
	Bovijn J et al.	4.169	0.842	0.005	0.755	4.914	0.860
IBS	FinnGen	69.555	0.392	0.003	0.880	71.601	0.379
	UK Biobank	32.752	0.288	0.010	0.865	35.003	0.292
CRC	FinnGen	34.856	0.427	−0.001	0.965	36.786	0.450
	UK Biobank	3.733	0.880	0.118	0.211	4.599	0.893
NAFLD	FinnGen	16.801	0.267	0.041	0.459	19.256	0.267
	UK Biobank	6.666	0.353	0.059	0.834	9.050	0.390
ALD	FinnGen	10.072	0.122	0.100	0.095	12.691	0.183
LC	FinnGen	16.667	0.825	−0.018	0.187	0.292	0.852
	UK Biobank	4.096	0.536	0.012	0.809	4.554	0.726
	Bovijn J et al.	28.230	0.297	−0.006	0.510	30.553	0.307
CP	FinnGen	6.384	0.973	−0.042	0.263	7.288	0.976
	UK Biobank	1.463	0.691	−0.044	0.728	2.412	0.707
	Bovijn J et al.	12.890	0.535	−0.010	0.565	15.209	0.481

### 3.3 Replication results

**Figure 4:**
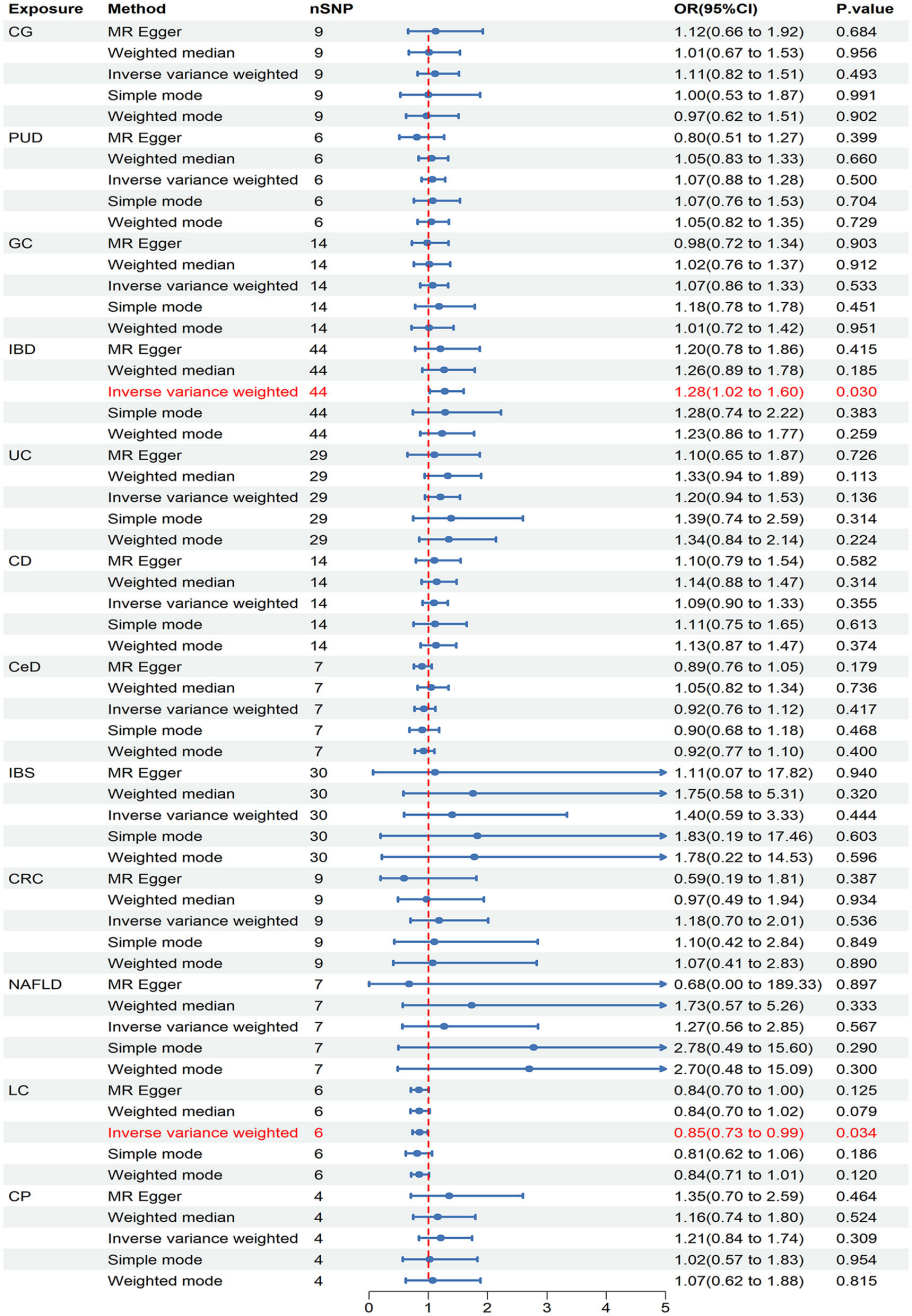
Causal estimates of chronic gastrointestinal diseases on ED in UK Biobank. CI, confidence interval; CG, chronic gastritis; PUD, peptic ulcer disease; GC, gastric cancer; IBD, inflammatory bowel disease; UC, ulcerative colitis; CD, Crohn's disease; CeD, celiac disease; IBS, irritable bowel syndrome; CRC, colorectal cancer; NAFLD, nonalcoholic fatty liver disease; LC, liver cirrhosis; CP, chronic pancreatitis.

In the initial stage of validation, our analysis focused on ED data obtained from the UK Biobank study. Our IVW analysis revealed that IBD (OR = 1.279, 95% CI = 1.024–1.579, *P* = 0.030) and LC (OR = 0.849, 95% CI = 0.730–0.987, *P* = 0.034) may increase the risk of ED (Figure 4). There were no significant findings of pleiotropy or heterogeneity (Table 1). Through leave-one-out analysis, no single SNP significantly influenced the estimated correlation was found. Additionally, the replication study in stage 2 did not find any gastrointestinal disorders that had an impact on erectile dysfunction (Supplementary Table 5).

### 3.4 Final causality of chronic gastrointestinal diseases on ED

**Figure 5:**
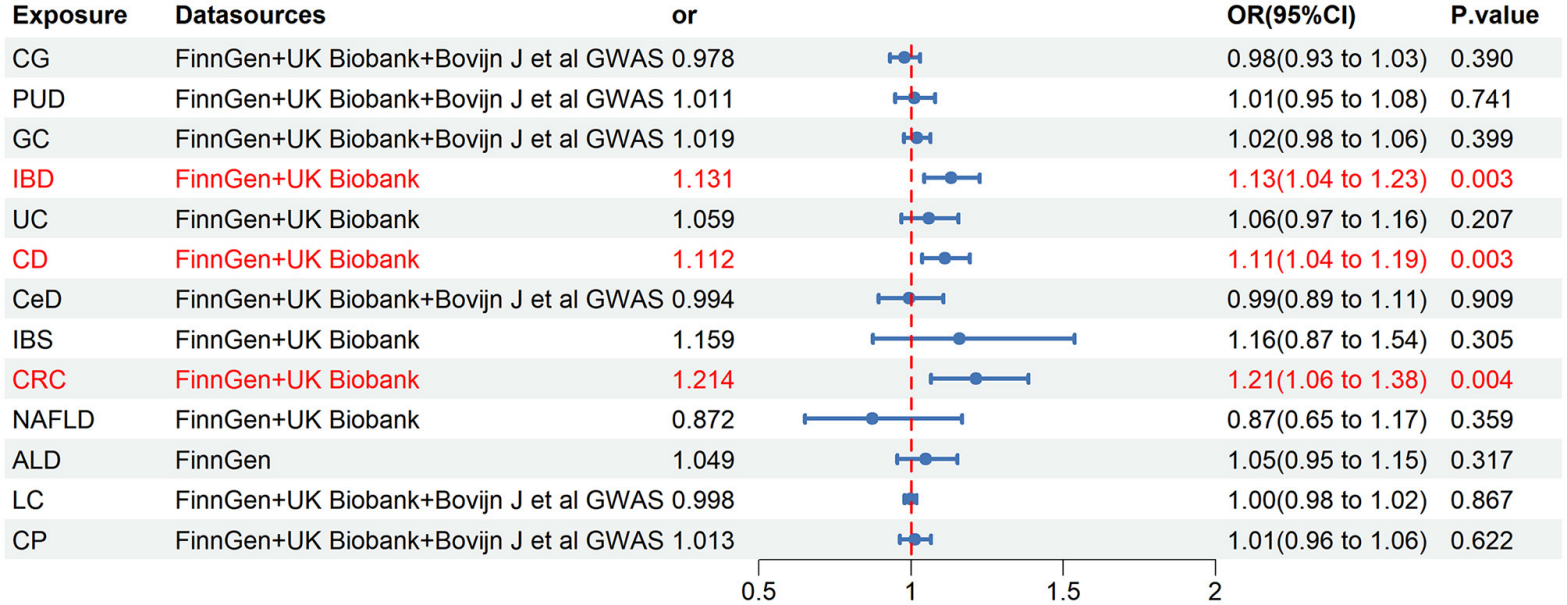
Final causality of chronic gastrointestinal diseases on ED. CI, confidence interval; CG, chronic gastritis; PUD, peptic ulcer disease; GC, gastric cancer; IBD, inflammatory bowel disease; UC, ulcerative colitis; CD, Crohn's disease; CeD, celiac disease; IBS, irritable bowel syndrome; CRC, colorectal cancer; NAFLD, nonalcoholic fatty liver disease; ALD, alcoholic liver disease; LC, liver cirrhosis; CP, chronic pancreatitis.

Our meta-analysis synthesized a minimum of two trustworthy MR analysis studies. However, only one reliable MR analysis result was available for the relationship between ALD and ED, thus the final determination of causality between ALD and ED was based on this single result. The results revealed a significant causality between IBD and ED (OR = 1.131, 95% CI = 1.043–1.226, *P* = 0.003), as well as a significant causal association between CD and ED (OR = 1.112, 95% CI = 1.037–1.193, *P* = 0.003) (Figure 5). Moreover, the pooled analysis indicated that CRC had a potential causal effect on ED (OR = 1.214, 95% CI = 1.065–1.385, *P* = 0.004) (Figure 5). Notably, our analysis did not find causality between other chronic gastrointestinal diseases and ED based on the combined results.

## 4 Discussion

The incidence of ED is associated with various risk factors, such as socioeconomic status, mental health issues, and physical health conditions (42). However, the relationships between chronic gastrointestinal disease and sexual function have been neglected.

The causal relationships between chronic gastrointestinal disease and ED were thoroughly explored in our recent study using MR analysis. The results of LDSC regression analysis indicated a significant forward genetic correlation between CD and ED. Potential forward genetic correlations were identified between IBD and UC with ED, whereas LC showed a negative suggestive genetic correlation with ED. The MR study revealed robust associations between IBD and CD with ED. Additionally, our findings suggested a possible causal relationship between CRC and ED. Overall, the study illuminates the potential influence of gastrointestinal health on erectile function.

Previous observational studies have faced challenges in establishing causal conclusions between IBD and ED (43). Confounding factors could potentially influence the results, and the possibility of reverse causation adds further complexity to determining causality. A prospective cohort study demonstrated that a significant proportion of male IBD patients reported suffering from ED (44). Another systematic review estimated the prevalence of ED in approximately 27% of all patients with IBD (45). Furthermore, a cross-sectional study emphasized an increased risk of ED associated with IBD, underscoring the importance of addressing sexual health issues in this specific patient cohort (46). Conversely, another cross-sectional study involving 119 male patients with IBD found no significant association between IBD and ED (47). In conventional observational studies, the presence of numerous confounding factors may account for the disparate results. The MR analysis we used can effectively mitigate biases related to confounders like hypertension and diabetes, as well as biases related to reverse causality concerns (48). The combined findings of our study revealed a strong association between IBD and ED. Additionally, the evidence derived from the study suggested that CD presented a significant risk for developing ED. Our MR study is consistent with a recent study. Our investigation has the advantage of examining the relationship between IBD and ED by analyzing data from two large databases—the FinnGen and the UK Biobank study. By combining the findings from both datasets through meta-analysis, we were able to provide a more comprehensive conclusion. This methodology enhanced the reliability of our MR findings. In our MR analysis, CD was recognized as a contributing factor to ED, whereas UC did not exhibit any correlation with ED. This discrepancy may be attributed to genetic variations between CD and UC. Further research is required to validate this hypothesis (14). A potential mechanism underlying the association between IBD and ED involves hypogonadism and microvascular endothelial dysfunction as a result of chronic inflammation (46, 49).

Previous prospective cohort studies have suggested an association between CRC and ED, although this connection remains inconclusively verified (50). Our study discovered a possible connection between CRC and ED by analyzing ED data from the FinnGen database. This correlation was confirmed through a comprehensive meta-analysis. Additionally, our research did not reveal any significant indications of pleiotropy or heterogeneity, indicating a robust and reliable association between CRC and ED. Pelvic pain, nerve damage, and fibrosis of the genital tissues induced by CRC could represent potential mechanisms underlying this correlation (51).

Associations between LC and ED were observed in the UK Biobank, but not found in the FinnGen and Bovijn J et al.' GWAS. This discrepancy could be due to differences in gene pool composition between the three databases (52). The observed discrepancy may also be influenced by the insufficient statistical power in the replication stage 1, as the sample size of ED cases in UKB was much smaller than in FinnGen and Bovijn J et al.' GWAS. Additionally, our method of selecting IVs may not entirely eliminate weak instrumental bias, which could also impact the differences in results. However, the combined result confirmed no causal relationship between LC and ED, indicating the reliability of the result.

A prior observational study indicated a relationship between ALD and ED (53). However, our MR research did not reveal a direct causal connection between ALD and ED. This evaluation of causality was exclusively carried out in the FinnGen database due to restricted GWAS data availability. Further investigation is necessary to provide a more in-depth comprehension of the potential relationship between ALD and ED.

Previous observational studies have suggested relationships between CG, PUD, GC, CeD, IBS, NAFLD, CP, and ED. However, these connections were not supported by the findings of this study. Causal relationships between CG, PUD, GC, CeD, IBS, NAFLD, CP, and ED cannot be definitively excluded in our MR study due to limited evidence.

Our MR research provides significant advantages. This study stands out as the first comprehensive investigation in this area, distinguishing it from previous observational studies. By using MR analysis, we were able to address potential biases, enhancing the credibility of our findings. Moreover, our selection of GWAS data primarily from European populations helped to minimize population structure bias. In addition, we undertook an additional measure by performing a Meta-analysis on three sources of GWAS data to strengthen the reliability of research findings. Furthermore, sensitivity analyses were applied to address issues such as horizontal pleiotropy and potential statistical biases.

However, acknowledging the limitations of our research is crucial. Firstly, the use of data from various sources to enhance result confidence might have introduced heterogeneity in the meta-analysis, attributable to the differing definitions of ED cases among various databases. Secondly, although MR analyses typically require non-overlapping participants between exposure and outcome samples, we were unable to determine if there was any sample overlap in our study. Nonetheless, we minimized potential bias from sample overlap by excluding IVs with *F* statistics < 10. Thirdly, insufficient suitable data prevented us from conducting sex-stratified analyses and assessing the severity and type of ED, potentially introducing biases. Additionally, as the SNPs examined were only of European descent, we cannot apply our findings to other populations. As indicated by the European Association of Urology (EAU) Guidelines for Male Ailments, the incidence of ED is steadily on the rise across all racial groups (54). Hence, further investigation is warranted to explore the causal link between chronic gastrointestinal disorders and ED across various racial demographics. Moreover, even though we made an effort to account for gene pleiotropy in our analysis, biases may still be present. Consequently, future research endeavors must include larger sample sizes in MR studies or RCTs to confirm the results of our study.

In conclusion, a causal relationship exists between IBD, CD, CRC, and ED. IBD, CD, and CRC may elevate the likelihood of developing ED.

## 5 Conclusion

In summary, IBD and UC show potential forward genetic correlations with ED, while CD has a significant forward genetic correlation with ED. Additionally, LC demonstrates a suggestive negative genetic correlation with ED. MR analysis indicates significant causal relationships between IBD and CD with ED, while CRC may have a potential causal effect on ED. These results underscore the importance of considering the impact of chronic gastrointestinal diseases on ED in clinical practice.

## Data availability statement

The datasets presented in this study can be found in online repositories. The names of the repository/repositories and accession number(s) can be found in the article/Supplementary material.

## Author contributions

XZ: Writing – original draft, Software, Methodology, Investigation, Formal analysis, Data curation, Conceptualization. LT: Writing – review & editing, Supervision.
